# Decision-making process in game sports: what do top-level players think of current research?

**DOI:** 10.3389/fspor.2025.1653834

**Published:** 2025-09-26

**Authors:** Lukas Magnaguagno, Damian Beck

**Affiliations:** Institute of Sport Science, University of Bern, Bern, Switzerland

**Keywords:** expert performance, deductive thematic analysis, gaze behavior, information integration, decision determinants

## Abstract

**Introduction:**

Excellent performance in game sports is largely determined by a functional decision-making process which integrates different perceptual-cognitive skills. This paper aims to bridge empirical and practical knowledge by exploring top-level players' subjective experiences in decision making.

**Methods:**

A semi-structured interview guide was developed and informed by existing decision-making literature including three categories (i.e., gaze behavior, information integration, and decision determinants). Each Switzerland's most successful male and female player from five different game sports (i.e., beach volleyball, floorball, handball, ice hockey, and soccer) participated in single interviews, focusing different aspects within each of the three categories. Verbal data were analyzed using a deductive thematic analysis after which top-level players' perspective was compared against the selected theoretical concepts and empirical findings.

**Results:**

The data analysis showed that top-level players use gaze anchor and foveal spot for peripheral vision, anticipate based on a weighted combination of kinematic and different contextual information, and decide predominantly intuitively by applying if-then automatism.

**Discussion:**

The present findings reflect well-examined factors of the decision-making process and uncover specifics relevant to top-level players but underrepresented in the literature such as decisional relevance when using peripheral vision, or tactical strategies influencing decisions. Therefore, our results provide player-perspective information underlying the decision-making process in complex game situations as well as valuable recommendations for applied practices.

## 1 Introduction

In game sports, perceptual and cognitive abilities have been identified as key factors contributing to superior performance within the multidimensional spectrum of potential attributes (1). Perceptual-cognitive skills refer to environmental information, domain-specific knowledge, and motor capabilities (2–4). Of particular interest in the realm of game sports are perceptual-cognitive skills within behavioral control (5). This involves an athlete's ability to use information from the current situation, apply knowledge to anticipate, select functional actions from numerous possibilities, and execute the chosen goal-directed action (6). Existing research has attempted to define what is at the heart of superior performance regarding (i) perception, (ii) information integration, and (iii) decision determinants (7).

First, perception, defined as capturing data at a sensory level, depends on the use of multiple sources of information (8). Research using eye-tracking technology to examine gaze behavior, wherein visual information is considered the most important sensory input (9), has provided an enhanced understanding of the gaze strategies used by expert performers (10). Specifically, experts are able to perceive rich chunks of information from pertinent cues by using systematic visual search patterns that can be adapted according to the changing demands of the task (11). In addition to gaze location, peripheral vision is important for the decision-making process in game sports because where players look may not always be where their attention is located (12). In this regard, research suggests that experts use peripheral vision more effectively compared to their counterparts (13). Furthermore, three functional differences between types of gaze behaviors which optimize the use of peripheral vision have been identified; the foveal spot is focused on a relevant cue to process detailed information, with attention ranging in width, the gaze anchor is located between relevant cues to distribute attention to them, and the visual pivot is located between or on a relevant cue to monitor the environment and select the next fixation point (14). However, to complement the insights derived from recorded eye-tracking gaze data and to further deepen our understanding of the perceptual processes underlying expert performance, qualitative investigations involving truly elite athletes are strongly recommended—especially with regard to their focus of attention (15).

Second, information integration refers to the use of a variety of information sources, such as perceived cues during a game. In this context, Bayesian integration is a framework which can help explain and investigate the anticipatory decision making of players (16). According to Bayesian integration, players use kinematic and contextual information to combine multiple incoming sensory inputs with previously acquired task-relevant knowledge (16). Whilst kinematic information includes equipment information (e.g., tennis racket), ball flight, and postural cues as well as body motion of other players (17), contextual information includes all non-kinematics: situational (e.g., players' positioning on the court), external (e.g., weather conditions), and player-specific (e.g., opponent's preferences) information (18). Players, who successfully combine and weigh these information sources, are able to minimize the uncertainty that accompanies decisions in game sports and make more accurate estimations of their current state as well as situational probabilities (19). Specifically, the reliance on contextual information becomes dominant before or in early stages of a movement execution. Then, when actions unfold, kinematic information is weighed more heavily (20). This suggests that individuals rely more on the information source that is associated with greater certainty (21) and that kinematic information—available later (e.g., postural cues) of a movement execution—is used to confirm, update, or override previous estimations (22).

Notably, the efficient use of multiple incoming sensory inputs as well as contextual knowledge depends foremost on the knowledge of the relationship between information sources and event outcomes (22) and the players' level of domain-specific expertise (23, 24). Moreover, different acquisition processes (i.e., implicit vs. explicit) need to be considered with respect to the reduction of uncertainty through acquired contextual knowledge. For example, recent findings revealed that non-expert players' anticipatory decision making improved more from an explicit provision of contextual information, whereas expert players' decision making improved when the contextual information was self-generated (25). More precisely, the information gain of explicitly provided contextual information seems to be grounded generally on the learner's level of expertise, the information (un)certainty as well as the amount of accumulated self-generated prior knowledge (26), and on a more differential perspective on personality factors such as visuo-spatial working memory (27).

Third, the majority of research on decisions has been conducted from a cognitive perspective that attributes players' superior performance to internal mental representations based on pronounced task-specific information and the subsequent facilitation of cognitive processes (28). One major finding is that decisions depend on the typicality of the situation and the availability of time; while a familiar environment or high time demands evoke an intuitive response, a consciously elaborated process is afforded in atypical environments and less time pressure (11, 29). In this respect, the existing research suggests that (i) highly skilled players rely on the first generated option (30), and (ii) tactics are a key cause of superior decisions as they help to scaffold the game (11). In addition to tactics and experience, emotional regulation (e.g., anxiety) influences performance because emotions can affect efficient beliefs and attentional style, which in turn interactively influences information processing (31). Finally, making decisions is not limited by the execution of the action but by the players' analysis of their behavior; decision-making skills rely on the effective development of slow, deliberate thinking and the transfer into the applied tactical knowledge (32).

The exploration of experiential knowledge of truly elite players and coaches by qualitative methods as an approach to understanding the entire decision-making process has largely been neglected in research, even though it is a fruitful and holistic approach to depict the complexity of perceptual-cognitive factors (18). To the best of our knowledge, only a few qualitative studies with high-skilled players on specific aspects of the decision-making process in a game sport have been conducted to date (17, 18, 33–36).

Using an open-coding analysis approach, Carboch et al. (17) and Vernon et al. (36) focused on a temporal model of information integration to demonstrate the sequence and interactions of kinematic (ball toss) and contextual information (score, surface, weather, and player handiness) by inductively examining professional tennis players. Schläppi-Lienhard and Hossner (18) conducted an inductive content analysis to identify opponent specifics, opponents' movements, and situational context as factors. Assessing the aspects perception and information integration, they found that experts' decision making in beach volleyball defense relies on the aggregated aspects of visual information (i.e., situational context, and opponent's movements) as well as domain-specific knowledge (i.e., opponent specifics, external context, and intuition). Contrastingly, Macquet and Fleurance (35) explored decision determinants by inducing eight types of decisions (e.g., put pressure on an opponent) from self-confrontation interviews with high-level badminton players during the course of a rally to capture the optimal conditions for ending the rally successfully. Their findings suggest that making decisions depends highly on understanding the immediate situation. Besides the aforementioned studies within net/wall games, there are only two qualitative analyses of invasion game players. Lenzen et al. (33) conducted an inductive analysis of self-confrontation interviews and demonstrated that decisions of high-level female handball players included perception and information integration (i.e., knowledge, expectations, and contextual elements. Similarly, Levi and Jackson (34) used an inductive method to examine professional soccer players' decision making during a match by concentrating on information integration and revealed four contextual aspects (i.e., personal performance, score status, momentum, and external/coach instructions) and three static contextual aspects (i.e., match importance, personal pressures, and preparation).

Existing research on the experiential knowledge of high-skilled players has provided interesting insights by taking an exploratory approach and focusing on a single game sport as well as particular aspects of the decision-making process. As a result, however, there are currently three approaches that are lacking when examining truly elite players in game sports: (i) a comprehensive consideration of the entire decision-making process rather than particular aspects; (ii) an overarching sample of game sports assessed using the same method to enable representative conclusions to be drawn; and (iii) a reflective insight into existing theories and empirical evidence, achieved through the use of a deductive approach. Therefore, this paper aims to contribute to the qualitative scholarship by examining top-level players' perspectives from five different game sports regarding gaze behavior, the use of different sources of information according to the Bayesian theory, and the way decisions are actually made. In addition to developing the existing scholarship regarding relevant theories, we aim to provide coaches and players with tangible knowledge regarding the development of decision-making skills.

## 2 Method

### 2.1 Participants

One Swiss female and one Swiss male top-level player from each of five different game sports (i.e., beach volleyball, floorball, handball, ice hockey, and soccer) participated in this study (P01–P10). The players' mean age at the time of the interview was 32.4 (SD = 5.7 years; age range: 25–43 years) and they had been competing at the adult level for 14.9 years on average (SD = 4.8 years). All players had several years of experience at the highest international level and were world-class. They agreed that their names and basic background information about their careers can be reported (for details, see Table 1). Specifically, we used Swann, Moran & Piggott's (37) heuristic device to classify the expert sample. This classification system includes three main themes to judge the validity of elite athletes within their sport (i.e., highest standard of performance, success at highest level, experience at highest level), and two further themes to determine validity across sports (i.e., national and global competitiveness of sport). For each theme, a score from 1–4 is achievable. By using the proposed equation, elite athletes are allocated to one of four categories: semi-elite (1–4), competitive elite (4–8), successful elite (8–12), and world-class elite (12–16). However, with the proposed equation, only a total score of 13.33 is possible. Consequently, we adjusted the equation so that experience at highest level was not halved and therefore the score of 16 points could be achieved. The scores of all ten recruited players ranged from 12–16 points (Mscore = 14.4, SD = 1.5).

**Table 1:** Name, type of game sport, experience in years and main achievements of the ten top-level players.

Name	Game sport	Experience	Main achievements (as per July 2024)
Anouk Vergé-Dépré	Beach volleyball	15 years	1-time Bronze Medal Olympic Games, 1-time 4th Place World Championship, 1-time Gold Medal World Tour, 1-time European Champion, 4-time Swiss Champion
Marco Krattiger	Beach volleyball	12 years	2-time 5th Place European Championship, 1-time Continental Cup Winner, 1-time King of the Court Champion, 3-time Swiss Champion
Corin Rüttimann	Floorball	16 years	2-time Silber Medal World Championship, 4-time Bronce Medal Word Championship, 4-time Swiss Champion, 2-time Swiss MVP, Record Scorer National Team
Mathias Hofbauer	Floorball	23 years	7-time Bronze Medal Word Championship, 2-time Topscorer World Championship, 3-time Allstar World Championship, 10-time Swiss Champion, 3-time Swiss MVP
Kerstin Kündig	Handball	17 years	1-time German Champion, 1-time German Cup Winner, 2-time Swiss Champion, 2-time best Swiss Player, 3-time Swiss MVP
Andy Schmid	Handball	20 years	1-time EHF Europe Cup Winner, 2-time German Champion, 1-time German Cup Winner, 2-time Swiss Champion, 5-time German MVP, 2-time Swiss MVP, Record Scorer National Team
Alina Müller	Hockey	7 years	1-time Swiss Champion, 1-time Bronze Medal Olympic Games, 1-time Best Forward and Allstar Olympic Games, No 3 Draft Pick PWHL, 3-time Best Swiss Player
Nico Hischier	Hockey	9 years	1-time 2nd Place World Championship, No 1 Draft Pick NHL, Allstar NHL, Rookie of the Year CHL, Trophée Michel Bergeron, E. J. McGuire Award of Excellence
Lia Wälti	Soccer	14 years	1-time English Champion, 1-time German Indoor Cup Winner, 1-time Swiss Champion, 3-time Top 100 Women's Players, 2-time Swiss Player of the Year
Xherdan Shaqiri	Soccer	16 years	2-time Champions League Winner, 1-time English Champion, 3-time German Champion, 2-time German Cup Winner, 3-time Swiss Champion, 2-time Swiss Player of the Year

### 2.2 Interview guide

In preparation for data collection, a semi-structured interview guide was initially developed by the first author, whose research and teaching activity has been on decision making in game sports for several years, with a focus on eliciting players' subjective experiences and reflections on existing research surrounding the decision-making process. The interview guide was then adapted based on discussions with the co-author as well as other researchers. Specifically, each interview began with a series of openly framed questions such as “Explain how you make a decision in the game”. Later in the interview, questions were introduced to obtain reflective insights of the three pre-determined categories within the decision-making process (i.e., gaze behavior, information integration, and making decisions).

The interview process of each categorical section included practice-oriented questions relating to existing research, inviting the player to comment on their extensive experience at the top level in relation to the theoretical and empirical work. Obviously, the participants were unfamiliar with the terminology employed in existing theoretical and empirical studies (e.g., kinematic information). Consequently, the meaning of any scientific terms used in the interview was summarized accurately to the interviewee before or after asking a respective question. However, the starting point for each category was a rather abstract description of a situation according to the categorial topic by the interviewer, and participants had to specify the situation within their own game sport. Followingly, they were indirectly asked a question about their subjective beliefs regarding a research finding. This unique approach provided a general introduction to the categorical topic, enabling the subsequent elaboration of the participants' individual behavior in the particular situation. Regarding contextual information, for instance, players were asked: “Let's assume that in a specific situation, the teammate or opponent repeatedly displays a certain behavior. Can you imagine a specific situation involving a teammate or opponent? Please describe the situation. If top-level players are either explicitly informed (instruction from the coach) about this behavior or learn it themselves through experience, what do you expect, which players make better decisions, and why? What about you? In which condition would you make better decisions?”

To ensure that the language of the questions was accessible to the participants and that this process still encouraged subjective beliefs, the interview guide (see Supplementary Material) was tested with three pilot interviews and, if necessary, adapted before data collection.

### 2.3 Data collection

Following approval of the study protocol from the ethics committee of the university faculty, players were invited to take part in interviews. The first author was able to contact select players through a personal network, while others were contacted through their management or respective federation. After willingness to participate in our study was confirmed, face-to-face interviews were scheduled with the first author according to each player's preference. Prior to the interviews, each participant reviewed and signed informed consent forms, where players were guaranteed that the specific information they shared during the interview would not be linked to their names. To this end, the participant's code (i.e., P01–P10) is anonymous and players statements were also made anonymous by masking or altering any identity revealing information. Then, when it came time to conduct the interviews, we ensured that all participants addressed each topic, while ensuring that the sequence of the questions was flexible and follow-up questions could encourage players to elaborate their responses. The recorded interviews were held in German, the native language of all the participants, and lasted between 48 and 75 min (M = 59.1 min, SD = 7.3 min). At the end of each interview, athletes were invited to discuss any points they felt had been neglected.

### 2.4 Data analysis

Recorded interviews were transcribed verbatim and each participant was given a random identifier such as P2. Transcripts were analyzed using a deductive thematic analysis to understand how existing research compare to lived experience of superior performance in game sports. Using a deductive method allowed us to search for elements in participants' interviews which resonate with or are in opposition to existing research. The analysis followed the seven-step procedure from Mayring and Brunner (38). First, a structured categorization matrix was developed based on existing theories and empirical evidence in the realm of the decision-making process in game sports (39). In the second step, the coding guide as a primary instrument was created based on the matrix, including operational definitions and typical text passages serving as anchor examples for each subcategory within the respective category (40). Third, half of the data (i.e., first five interviews) was reviewed for content and coded according to examples of the predetermined subcategories (41). In the case that relevant data did not connect to a pre-determined code, new codes were developed and added to the coding guide to ensure factors relevant to players were not being neglected (Miles, Huberman, & Saldaña, 2020). In the fourth step, the second author joined the analysis process and reviewed the categorization matrix for comprehensiveness and consistency (see Table 2). The two authors revised elements as necessary and finalized the coding guide. Fifth, the entire data set was coded in correspondence with the finalized coding guide by the first authors, meaning that codes of the first half were adjusted if necessary. In the sixth step, the second author fully coded all data to improve validity. To this end, all coded data units were extracted from the interviews, randomly ordered, and existing codes were removed to limit bias. As interpretational differences within a data unit might still occur systematically, a first comparison was made after the second author coded the first 99 data units (=15%). The analysis of the intercoder reliability revealed a variance-weighted average of Cohen's kappa of 0.58 [95% CI = (0.48; 0.68)] for the categories, and 0.58 [95% CI = (0.50; 0.66)] for the subcategories (42). To unify the interpretation of a data unit, the first author reasoned his categorial choice for all data units with different codes. After the second author had read the first author's remarks, he coded the remaining 563 data units, and followingly, the intercoder reliability was calculated for the entire data set (43). Finally, a variance-weighted average of Cohen's kappa of 0.64 [95% CI = (0.60; 0.67)] for the categories, and 0.54 [95% CI = (0.51; 0.57)] for the subcategories was found, implying a substantial and moderate agreement, respectively (44). Intercoder disagreements were analyzed and discussed until a triangular consensus between both raters was obtained. More precisely, for units with different codes, the first rater indicated whether he (i) agreed with the coding of the second rater and simultaneously disagreed with his own, (ii) saw both codes as valuable, or (iii) disagreed with the coding of the second rater. If (i) applied, the code of the second rater was used. In case (ii), both codes were included in subsequent analyses. In case (iii), the first rater explained why his code decision was more appropriate than that of the second rater. Followingly, the second rater either agreed with the first rater's reasoning or had the opportunity to justify his own coding. In a few cases where no consensus could be reached through further discussion, both codes were implemented. In the seventh step, the coded data units within each subcategory were clustered into different aspects so that the collective players' beliefs could be extracted and related to the corresponding subcategory.

**Table 2:** Categories and subcategories of the finalized matrix.

Categories	Subcategories
Gaze behavior	Relevance of perception, foveal spot, gaze anchor, visual pivot
Information integration	Situational information, external information, player-specific information, kinematic information, information weighting, information uncertainty, information acquisition
Decision determinants	Decision mode, decision causes, influencing factors, decision (self)analysis

## 3 Results

**Figure 1:**
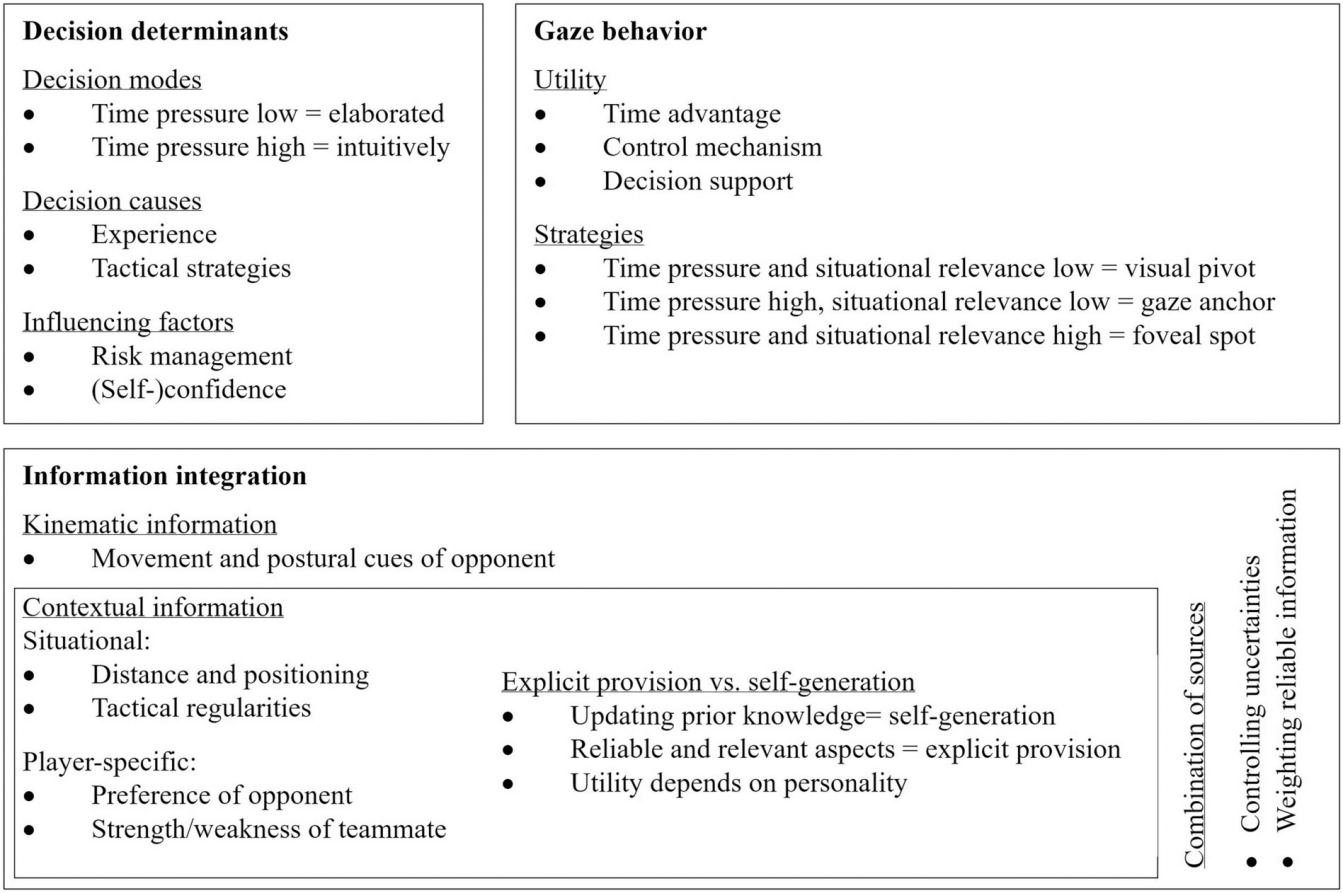
Top-level players' perspective on the three categories (i.e., gaze behavior, information integration, and decision determinants) of superior performance based on deductive thematic analysis.

In our analysis, we extracted 662 data units, which resulted in a total of 715 coded statements as some data units were assigned to multiple subcategories. With respect to the categories, most statements (=348) were related to decision determinants, followed by information integration (=261) and gaze behavior (=106). The chosen quotes were translated to English using ChatGPT-4.0 and edited for readability where necessary. Additionally, a few parts had to be generalized to maintain participant anonymity. These changes are indicated with square brackets. The chosen quotes below represent the players' shared perspectives regarding a specific aspect or subcategory. The following result section is organized according to the introduced categories (i.e., gaze behavior, information integration, and decision determinants). The synthesized aspects related to (sub)categories resulting from deductive thematic analysis are depicted in Figure 1.

### 3.1 Gaze behavior

This category includes the utility of visual perception and the gaze strategies used for peripheral vision. In addressing the former, three general functions could be divided. First, the importance of capturing visual information about the positions and movements of opponents and teammates as well as free space on the pitch is related to a time advantage [P07: “Of course, you have to look beforehand to see where the players are open. That helps a lot in making a decision. If you don't look at the players beforehand and then get (the playing device), it takes too long to make the right decision.”]. Second, visual perception functions as a “control mechanism” for beforehand anticipated situational probabilities because, although players believe to know how the situation will play out, they cannot be certain (P03: “I assume that, but I don't know for sure yet if that will happen 100%. That's why I still have to check if the opponent has indeed reacted that way, if my teammate has actually taken this running path with this timing as I had imagined. That's why these checks are necessary.”). Third, visual perception is used as decision support to finally choose a specific action. Therefore, the attention is directed to pertinent cues.

Regarding gaze strategy and the functionality of peripheral vision, players struggled to explicitly define the type (i.e., foveal spot, gaze anchor, and visual pivot) they use in a specific situation and instead reported a certain feeling for their strategy (e.g., gaze location). In general, gaze anchor and foveal spot compared to visual pivot were described as predominant strategies. Additionally, two conditions were described as determining the type of gaze behavior for optimizing the use of peripheral vision: the available time before a decision must be made, and the relevance of the decision. As most of the game situations occur under high time pressure, players' statements revealed that there is no time to locate the gaze between or on a relevant cue to select the next fixation point. To monitor relevant cues, the gaze is rather fixed on a point in the environment (i.e., gaze anchor) or on an object (i.e., foveal spot). When asked about a fixation point, Player 5 said [P05: “That means the more directly I look at the goal, the more overview I have of what is happening on the left and right. (…) So, I position myself in such a way that I give my vision the chance to see the whole situation.”]. With respect to a foveal spot, a relatively broad attentional width was mentioned so that pertinent information in the periphery is detectable, particularly in game situations of high relevance (P08: “I think when you only have the goalie in front of you, or are really preparing for a shot on goal, then only the net and the goalie are in view. Yes, if there is still a player in position somewhere, so that you could make a pass, then I think you notice that, or you hope to notice it. But if you have decided that you are going to shoot, then you are very focused on the goalie.”).

### 3.2 Information integration

This category refers to kinematic information, contextual information, and their combination as means to facilitate predictive decisions. When it comes to kinematic information, participants primarily reported the use of movement and postural cues from their opponents. In this regard, pertinent body parts such as trunk, hand, or gaze direction varied depending on the game sport (for example, P10: “Yes, I think the run-up, so the direction of the run-up, the speed of the run-up, and then also the upper body, so the movement in the upper body.”). In contrast, ball and teammate were less often mentioned as relevant kinematic information.

Similar to kinematic information, the three types of contextual information varied regarding their influence on the decision-making process. While external information was only mentioned by two players, situational and player-specific information was reported by all players. Regarding the former, two main aspects emerged from the players' statements. The first was an emphasis on the perceived distance of their opponent (P09: “So distance in the sense of how far away my opponent is from me, do I have time to turn, do I have time to switch sides, or is she so close that I have to find a solution very quickly.”) and even more often position-related information (P02: “You recognize or learn to recognize which foot positioning corresponds to which change of direction.”). In the context of the second aspect, the decision-making process is affected by “tactical rules” regarding teammates and the opponent in a rather general way (P03: “For example, opponents usually run towards their own goal, and they often fall too deep. Clever players then stay a bit in the high slot. We talk a lot about countermovement, but most of the time you don't even need to counter-move; they run towards their own goal anyway.”).

When it comes to player-specific information, this subcategory can be distinguished in the context of the referred individual (i.e., opponent and teammate) and the attributed factor (i.e., preference, strength, and weakness). Generally, the use of contextual information about the opponent was mentioned more often compared to information about the teammate. Within the statements regarding the opponents, the analysis revealed that preferences were the most prominent aspect (P03: “I can, I have a certain prior knowledge that there is a high chance he will do that. Then it is easier for me, yes, to orient myself or to check in advance if it really happens that way, because I already assume it will happen, because I know it.”), followed by strengths and weaknesses of the opponent [P10: “(…) so we always discuss this in advance with the coach. Where the opponent's weaknesses are, where the opponent's strengths are, and how they might behave offensively as well as defensively, what we expect.”]. This last statement, as many others, demonstrates the interrelation of the three factors. A similar finding was uncovered in the statements regarding teammates. However, in this case, the most frequently mentioned factor was the strengths of the teammate.

Besides the interrelation of player-specific information, participants explained that decisions rely on a combination of kinematic and contextual information. Moreover, the two sources are used in a temporal order, meaning that players rely on situational probabilities based on contextual information in the early preparation stage, and then kinematic information serves as an additional source, for instance, to check if the situation unfolds as predicted (P06: “I would say, neither more nor less. They are just in two different time frames. So, I go there. Then I know: ‘Okay, she has these and those options,’ and then I can already eliminate other things. So, I prepare myself for three or four things. And then, in the moment itself, it is the information about how she approaches the ball or what the movement looks like.”). This “control mechanism” was mentioned as a way to handle uncertainties that are particularly inherent in contextual information. Furthermore, participants explained that weighting either kinematic or contextual information depends on the reliability of the source. More precisely, the action tendencies of our participants can be derived from prior knowledge in situations with high certain contextual information, but an openness to different situational probabilities has to be maintained when contextual information is less reliable (P02: “If it is my opponent, whom I have analyzed, know, etc., then it is game-specific. And if it is a random player, and you think, um, I have no idea what she is doing. I don't know, and then it's definitely movement-specific because then you just have to react to what is happening.”).

A closer look at the acquisition of contextual information shows that a mixture of explicit provision and self-generation is needed to enhance the players' decision making [P08: “(…) I like a mix. I enjoy watching videos and listening to what the coach considers optimal. But then I try different things and see what works best for me.”]. On the one hand, instructions of the most reliable and important contextual information are useful to be well prepared for the game (P01: “Yes, because you are prepared. Because you can practice it in training. Because you can already coordinate with your teammates ‘Hey, if she receives the ball, then I will attack her, and you can already position yourself there, then you will get the ball from me’.”). On the other hand, prior knowledge based on the instructions have to be updated by the experiences during the game, where contextual changes might occur at any time. In this context, self-generated learning was mentioned as beneficial for a more sustainable effect regardless of the information certainty (P05: “I do believe that everyone is an individual athlete in some way. When you set a goal for yourself and it works, it has, let's say, a greater impact than when someone tells you to do something and you do it, and it leads to success.”). However, besides the general positive effect of a combination of explicitly provided and self-generated contextual information, our analysis revealed that the acquisition is related to the players' type, meaning that some players prefer to have instructions and others tend to trust their own experiences (P07: “Yes, I think that's very difficult to say. Because everyone learns differently, everyone plays differently. I believe there are certain players who need this information, and there are certain players who see and notice it themselves.”).

### 3.3 Decision determinants

This category contains decision mode, decision causes, and influencing factors. Regarding the decision mode, decisions can rely either on an intuitive-unconscious or an elaborated-conscious process. In this context, the statements showed that the time availability to make a decision is the most determining factor which of the two processing modes occurs. Whilst intuitively deciding is used under high time demands (P10: “I think when the reaction time is short, or the perception time, yes, is short. When you have little time to perceive something and act on it. I believe then it is intuition.”), for an elaborated decision, a certain time frame is needed as, for instance, in a set play situation [P10: “Yes, for example, during a (…), I think about which player to target, so I have a lot of time to decide which player to target and at which position, and with what speed, or focusing more on precision or more on speed. And that is something you actually prepare for.”]. Generally, however, intuitive-unconscious decisions were predominantly described, but elaborated processing, when possible, is integrated into the entire decision-making process so that a main acting plan can be consciously generated before the situation unfolds. Participants described that intuitive adaptions are made to the plan as necessary [P06: “I often prepare a plan in my head and can then decide at short notice whether it makes the most sense or not, and then I would switch depending on my (…). Depending on what I observe from the opponent, what I perceive. Sometimes it's peripheral. Sometimes I notice that the ball is so fast or so poorly played, whatever the case. That certain solutions are no longer viable, and then you have to adapt and find other solutions (…).”]. In addition to elaborated if-then planning described in the quote above, intuitive decisions rely partially on unconscious if-then automatism, which is developed over experiences in similar situations and triggered by stimuli such as a movement of the opponent (P01: “I believe you train it so often that it becomes automated. So, in training, you constantly go through game situations. And then, when you train it numerous times, it becomes automatic, and you don't have to think about it anymore. So, if two players come at me, then I know, okay, I have these options. And then I have to choose one, and I usually do that based on the movements of the opponent.”). In this regard, the participants described a preference for the availability of only a few options to be able to make efficient decisions.

In the context of possible decision causes, two core aspects were mentioned. On the one hand, game experience is needed to achieve a high level of decision-making expertise. Particularly, this includes the involvement in similar game situations repeatedly so that a wide and fine-grained database of experience is developed (P01: “I believe that is again based on experience. I have experienced this situation many times, I know it works. And in that moment, the opponent might make a move, and then I know, okay, I can go in the other direction. But I know this direction has worked many times before because I have experienced it so many times.”). Additionally, participants explained that experience can be indirectly gained by watching matches or visualizing game situations. However, the database of experience leads to prior knowledge of kinematic and contextual information, which are used for efficient decisions [P07: “You do it, and from playing (…) for a long time, you know certain situations and then make a decision. But it's not a conscious thought process; it's natural. You get the (playing device) in the middle, and I know exactly that when I have it and get pressure from this side, I know, okay, now I can pass it to him. Now my winger should actually come there. I could pass it to him or try to place it in the open space or turn away myself, and these are just things that I feel I don't consciously decide.”]. On the other hand, tactics and strategies were reported as determinants for making decisions, meaning that decisions are sometimes made just according to tactical rules which were, for instance, communicated beforehand of a game [P08: “Of course, we have a rehearsed (tactic) or a system that we should execute in a similar way, and then when you see that this player is running this path, you can already rule out certain things and know, okay, we will now come out on this side. Yes, it just helps for me to decide what I want to do.”].

When it comes to other decision determinants, there were two aspects (i.e., game management and confidence, respectively) that we derived from the statements. Participants broadly mentioned game management, but generally related to the differentiation of decisions within the course of the game. Particularly, players described risk management as a crucial factor of their decision making (P06: “And the other thing is really recognizing those small moments when to take more risks and when to take less. I believe that the very good players manage that really well. They also don't let themselves be thrown off by one decision, by two decisions, or by mistakes made at the wrong moment.”). The second aspect, confidence, was reported either in a broader sense or in the context of the players' own skills (P05: “It is pure trust in me, it is such a fundamental trust in my strengths, just trust in that I can do it, that I have proven it a thousand times. And also not being afraid of making wrong decisions. I think that is a very, very important point.”).

Finally, participants described that they frequently analyze their decisions to improve future plays. However, analyzing decisions generally occurs in situations with sufficient time availability such as halftime breaks or after the game. As a valuable tool, video footage is used to support the analysis.

## 4 Discussion

The aim of the present paper was to provide a reflective insight on existing research by exploring players' experiences surrounding decision-making process in game sports. In contrast to previous studies, which have primarily focused on a single game sport and specific decision-making factors using an explorative approach, we sought to understand the subjective experiences of this phenomenon across a wide range of game sports to critically examine the bridge between theory and practice. To this end, we used existing theories and empirical evidence on gaze behavior, information integration, and decision determinants as building blocks for interviews with top-level players of five different game sports.

Within the category gaze behavior, three aspects (i.e., time advantage, control mechanism, and decision support) were identified, which describe the utility of visual perception. While previous research has shown that players in game sports use pertinent cues to support their decisions (45), experimental studies that examined either time advantages to inform subsequent actions or players' control mechanisms to check whether a situation unfolds according to their prediction are still lacking. As a consequence for practice, players' gaze behavior should not only be enhanced by the knowledge of and the attention to pertinent cues (6), but also through utility differentiation, enabling them to use visual perception to gain time advantages and validate their anticipation. Regarding the former, for example, a midfield football player's shoulder checks help to scan action options before she/he even has received the ball.

Beyond, in our analysis, we revealed that players' descriptions of gaze behaviors which optimize the use of peripheral vision varied depending on exogenous conditions of game situations such as time pressure and decisional relevance (46). Obviously, the former refers to the available time before a decision has to be made. Contrastingly, decisional relevance defines how important a specific decision is within the game. For example, a penalty kick in soccer is (mostly) of high decisional relevance, while a pass from the central midfielder to the winger at the touchline is of lower decisional relevance. In this context, players reported that they are only able to use visual pivot when time pressure and decisional relevance are rather low. This relates to previous descriptions of a visual pivot in the literature, which is defined as holding gaze on a location close to relevant cues and initiating saccades to these cues (14). However, low time pressure situations are rare in game sports, and thus the strategy of visual pivot, which involves frequent fixations from and to a specific location to select the next target, might be less relevant for superior performance than expected, especially because each saccade involves costs (e.g., the suppression of information processing). Contrastingly, based on our analysis, we argue that gaze anchor and foveal spot strategies are of higher importance for top-level players' performance. Specifically, players described that a gaze anchor is used under high time pressure and low decisional relevance, and a foveal spot is needed under high time pressure and high decisional relevance. These findings are in line with previous research, which has suggested that pursuing a foveal-spot strategy is appropriate in situations when processing information from the periphery could lead to less accurate information being derived from the current gaze location (14). Additionally, gaze anchor has been referenced in situations with high time pressure where costs associated with saccadic eye-movements are eliminated (14). Besides this accordance with previous research, our findings emphasize the importance of decisional relevance as a further exogenous condition. We believe that exogenous conditions, particularly decisional relevance, should be explored more explicitly in future studies when examining the functionality of different gaze behaviors. With respect to practical implications, coaches should design exercises that manipulate time pressure and decisional relevance, forcing players to develop a functional gaze strategy.

Regarding the category information integration, the top-level players reported that both information sources previously described in the literature (i.e., kinematic and contextual) are relevant to their decision-making process, supporting perfectly the Bayesian framework (16). As conceptualized by this approach, top-level players in game sports seem to use the combined information from the multiple incoming sensory inputs (i.e., likelihood) with the previously acquired task-relevant knowledge (i.e., prior) for a joint probability distribution to accurately estimate the current state (i.e., posterior) and thus to better predict how situations will unfold (19). More precisely, players explained that they begin by trying to predict upcoming events based on contextual information, where they make use of situational and player-specific information. When it comes to situational information, the players shared that distance to and positioning of other players as well as tactical regularities are relevant. While recent studies have revealed that distance (46) and positioning (23) affect the decision-making process in game sports, tactical regularities are underexplored in the research. Additionally, the players referred to two aspects of player-specific information (i.e., preference of opponents and strength/weakness of teammates) that are often intertwined and of relevance for their decision-making process. Enhanced anticipatory decision making based on preferences of opponents has been more extensively investigated (24) compared to strength/weakness of teammates (26), and to our knowledge, no study has examined the interplay between the two despite the relevance for superior performance. Additionally, we note that top-level players, except for the two participants in beach volleyball, did not mention external information, implying that these conditions have to be considered only in specific game sports; for weather conditions, see (18) and for surface conditions, see (36).

Players emphasized that predictions made based on contextual information help them focus on specific possibilities of action and allow them to have action tendencies before kinematic information is available. Afterwards, kinematic information is used to verify if the situation unfolds as predicted (see also the utility of gaze behavior), and the action tendencies can be executed as planned or other action plans become dominant. We found that top-level players' reports aligned with existing literature that describes how using contextual information helps players constrain action possibilities and kinematic information to decrease the number of relevant options (47). With respect to kinematic information, the players mentioned movements and postural cues of opponents as important sources of information, but did not generally indicate kinematics of teammates or the ball flight. With respect to kinematics of teammates, we assume that top-level players have a highly differentiated prior knowledge of them so that the associated uncertainty is almost completely reduced, and thus incoming sensory information has no additional informational value. The fact that players neglected the ball's trajectory leads us to suggest that its relevance depends either on the individual or the type of game sport. In regard to the latter, our suggestion is supported by a large number of studies that have examined the use of ball flight information with a focus on striking sports (48). In the present study, we did not include top-level players from net/wall games, which might explain the absence of such statements from participants. However, top-level players highlighted that the uncertainties are managed through a combination of contextual and kinematic information. This means that when uncertain contextual information occurs, the player's decision making relies more on kinematic information. Again, this is in line with the Bayesian framework, whereby players rely more heavily on the more reliable information source (21), implying that contextual information dominates earlier in a movement execution (20) and kinematic information is utilized afterwards (22).

Consequently, our practice recommendation is that players have to be enabled to accumulate significant task-specific prior knowledge of different contextual as well as kinematic information by respective on-field experience opportunities (6). Only then will players be able to weigh the information sources accurately and in combination with the tactical instructions of the coach.

When it comes to acquired contextual information, the top-level players described that they prefer explicitly provided as well as self-generated prior knowledge. More precisely, they explained that explicit instruction should be provided only for highly reliable and relevant aspects, whereas the self-generated acquisition of contextual information was described as consistently beneficial because it facilitates updating the prior knowledge during the game and is more sustainable. These findings are in line with recent research, which has suggested that experts improve their performance based on self-generated contextual knowledge (25). Previous research has also demonstrated that the perceived benefit of explicitly provided contextual information compared to self-generated prior knowledge depends on the certainty of the information as well as the players' possibility to generate the knowledge by themselves (26). Furthermore, top-level players see the utility of the acquisition type as a function of their personality, meaning that certain players are more inclined towards coach instructions compared to others. In this regard, future experimental research that examines the effects of information gain of explicitly provided contextual knowledge could benefit from considering external (e.g., information uncertainty) as well as internal factors (e.g., working memory capacity).

In our study, when it came to decision determinants, two decision modes (i.e., elaborated vs. intuitive) were described by our participants, a finding which is consistent with existing theories (11, 29). Regarding the elaborated decision mode, top-level players reported that they make elaborate decisions when the time pressure within the game situation is low. In these cases, one action plan is favoured before entering the game situation, and other action plans are kept alive to adapt to situational changes. It could be argued that this kind of if-then planning is related to the “less-is-more” effect as a reduction of action options seems to increase the functionality of the final decision (30). However, we prefer to link this finding to the Bayesian integration framework, which suggests that prior knowledge shapes the decision in the most likely direction without eliminating other actions (16). This interpretation of shaping the decision according to the probabilities of competing action options is further supported by the affordance competition hypothesis (49). Regarding the intuitive decision mode, which is the predominant way to make decisions because most situations in game sports are under restricted time constraints, players emphasized the use of if-then automatism. This means that their decisions are triggered by prior knowledge relating to a respective cue (see also the utility of gaze behavior) and the complexity of the situation is simplified (50). Similar to the elaborated decision mode, our findings pertaining to the intuitive decision mode appear to align best with Bayesian integration, as top-level players use the combination of incoming sensory information with prior contextual knowledge for if-then functions (16). Thus, the intuitive mode also seems to be connected to the affordance competition hypothesis (49). This leads us to suggest that if-then automatisms are based on considerably strong priors that make one action option pop out from others. Although our findings support the theoretical derivations of the two decision modes, there is a lack of empirical research in game sports that explicitly differentiates between elaborated and intuitive decisions and their specificities. The differentiation between elaborated and intuitive decisions should also be considered in the applied setting. Therefore, practice sessions have to be manipulated based on time constraints to foster if-then automatisms in highly regular situations and teach if-then planning in more structured situations.

Irrespective of the decision mode, top-level players' decisions are the product of experience as well as tactical strategies. With respect to the former, the players highlighted the importance of experiencing relevant game situations repetitively, including simply watching games, so that a sufficient data basis (i.e., prior knowledge) can be generated. According to Kahneman and Klein (29), experiences of high task-specificity are one out of three crucial conditions to develop intuitive decision proficiency. More specifically, this experience allows for a more accurate calculation of situational probabilities of how a game situation will unfold (19). Although the use of prior contextual knowledge for decision making has been intensively examined in the last decade, previous studies have neglected to include tactical strategies, which are assumed to help to scaffold the game (11).

Beyond the decision causes, top-level players distinguished two influencing factors. First, they emphasized that their decisions tend to be guided by risk management, implying that a change in the problem constellation—even if it is just one factor, such as the current score in the game—influences the decision (6). In this context, risk optimization means that several decisions exist to solve a given problem constellation, it is valuable to estimate the uncertainty connected to these decisions to consider the associated risks. Consequently, we argue that top-level players try to obtain an optimal trade-off between outcome-related costs and rewards and thus maximizing the utility of the decision outcome (8). One of the few studies that aimed to assess risk management in game sports showed that the integration of contextual and kinematic information is affected by judgment utility (51). Consequently, this influencing factor should be considered or at least controlled when investigating decision making in game sports. Second, participants reported that (self-)confidence, as a general disposition and regarding one's own skills, was important to their performance. Although the influence of different emotions on decision making is well-examined (31), only a few studies have explored the effect of (self-) confidence on the skills of students and revealed that self-efficacy might be a significant predictor of decisions in game sports (52).

The players' beliefs about the decision-making process in the current study complement existing research, challenge scientific theories as well as empirical evidence, and provide suggestions for future research in game sports, but there are two limitations that must be acknowledged. While all participants met our inclusion criteria as truly elite players in game sports, they might be influenced by the Swiss cultural training systems. The effect of different nations' playing styles, for instance, has been anecdotally reported in tennis (53). Additionally, the generalizability of the results is limited by sample size. Therefore, our conclusions, which are based on an explorative approach, should be verified by quantitative analyses. However, taken together, our findings lead us to confirm that scientific theories and empirical evidence have already addressed crucial aspects of the decision-making process. However, the interviews we held with top-level players of different game sports revealed previously unconsidered factors. First, studies that aim to experimentally assess the functionality of gaze behaviors and optimize the use of peripheral vision should focus on visual spot and gaze anchor by manipulating situations under different time pressure and, particularly, decisional relevance. Second, Bayesian integration is a reasonable framework to investigate the decision-making process in game sports. However, future research should explore the interplay between situational and player-specific contextual information together with kinematic information, particularly regarding their serial importance. Third, the utility of self-generated and explicitly provided contextual knowledge should be examined differentially as the gain seems to depend on personality factors. Fourth, we recommend that future research distinguish between elaborated and intuitive decision making by manipulating the time pressure to decide. Furthermore, tactical strategies as one key cause for superior decisions as well as relevant influencing factors (i.e., risk management and confidence) should be included so that their effect can be disentangled. Besides pinpointing future research directions, bridging empirical and practical knowledge also yields conclusions that can be applied by coaches. Summing up, we recommend that coaches enable the players to gain comprehensive on-field experience, (i) so that they learn to use functional gaze behaviors to direct their attention to pertinent cues, (ii) accumulate task-specific knowledge so that they are able to accurately combine and weigh several information sources, and (iii) acquire differentiated if-then automatisms.

## Data Availability

The raw data supporting the conclusions of this article will be made available by the authors, without undue reservation.
