# Supplementary material for: Decision-making process in game sports: what do top-level players think of current research?
Source: Front Sports Act Living. 2025 Sep 26;7:1653834. doi: 10.3389/fspor.2025.1653834 (PMC12512046; doi:10.3389/fspor.2025.1653834)
Supplement: Supplementary file 1 [file Datasheet1.pdf]

Interview guide

Opening

“Explain how you make a decision in the game.”

Follow-up questions:
- “What influences your decision-making?”
- “Why do you have the feeling that a decision will be the right one?”
- “What does a right or wrong decision trigger in you?”

Contextual information

Step 1: “Let's assume that in a specific situation, the teammate or opponent repeatedly displays a certain behavior. Can you imagine a specific situation involving a teammate or opponent? Please describe the situation.

If top-level players are either explicitly informed (instruction from the coach) about this behavior or learn it themselves through experience, what do you expect, which players make better decisions, and why?

What about you? In which condition would you make better decisions?”

Step 2: “Assuming all players have been explicitly instructed about this behavior, what do you expect and why: The explicit information is more useful if this behavior is almost always (80% of the time) or only most of the time (60% of the time) exhibited?

What about you? In which case would you benefit more?”

Follow-up questions such as “Under what conditions is explicit instruction by the coach on player-specific information helpful?”

Step 3: “What player-specific information do you generally use when making decisions?”

Follow-up questions such as “Based on what, do you expect the next action of a teammate or opponent to be?”

Information integration

Step 1: “In addition to player-specific information, kinematic information can also be used when making decisions. Can you think of a specific situation in which both types of information are important? Please describe the situation.

How do you expect top-level players to rely on the two types of information for their decision-making behavior in this situation, and why?

What about you? How do you rely on these types of information?”

Step 2: “Assuming it is a situation where there is hardly any time to make the decision. Can you imagine such a situation? Please describe the situation. What do you expect and why: The decision is made on the basis of player-specific or kinematic information?”

Follow-up questions such as “Are there situations where the other type of information is more useful?”

Step 3: “Which of the two types of information do you generally prefer when making decisions?”

Follow-up questions such as “Why is this type of information more important to you?”

Gaze behavior

Step 1: “Imagine a specific situation that you often encounter in a similar way when making decisions. Please describe the situation.

If the direction of players' gaze is recorded in this situation, what do you expect, where do top-level players look, and why?

What about you? Where would you look?”

Step 2: “Assuming a situation in which many different people (teammates and opponents) influence the decision. Can you imagine such a situation? Please describe the situation. What do you expect top-level players to look at and why: (1) The gaze is fixed on one place and the surroundings are perceived out of the corner of the eye, (2) the gaze jumps back and forth between several places or (3) the gaze is fixed on one place, but the surroundings are not perceived?

What about you? Which of these three strategies would you apply?”

Follow-up questions such as “Are there situations in which the other two strategies make sense?” or “What influence does the available time have?”

Step 3: “What information do you generally perceive visually when making decisions?”

Follow-up questions such as “Why is this information particularly useful for you?”

Decision determinants

Step 1: “Earlier, you described a specific situation in which you often make decisions. Can you still picture this situation?

When top-level players are asked whether they are aware of what they are deciding in this situation at the moment of the decision or whether they do it intuitively, what kind of answer do you expect and why?

What about you? Are you aware of your decision, or do you decide intuitively?”

Follow-up questions such as “Under what conditions is a conscious or intuitive decision possible?”

Step 2: “Assuming top-level players are not given help to make the decision, or they are given if-then help (if x happens, choose y). What do you expect players to do better with or without help in making decisions, and why?

What about you? Do you feel that your decisions are partly based on if-then plans and why?”

Step 3: “How and when do you generally think about your personal decision-making behavior?”

Follow-up questions such as “What consequences do you draw from these thoughts?”

Closing

“What do you think is the most important thing overall when making decisions?”
